# Saints’ mobility and confinement: deconstructing Byzantine stories of (fe)male ascetics and monastics

**DOI:** 10.1111/emed.12643

**Published:** 2023-06-22

**Authors:** Christodoulos Papavarnavas

**Affiliations:** ^1^ Austrian Academy of Sciences

## Abstract

This article investigates stories of holiness which have ascetics or monastics as their hero(in)es and which develop based on a careful interlocking of two concepts: wanderings in urban or desert environments and self‐confinement in enclosed or secluded spaces. Through a close reading of two saints’ *Lives* (i.e., the *Life of Mary of Egypt* and the *Life of Matrona of Perge* ) dating to the early and middle Byzantine periods, the present analysis uncovers the tripartite relationship between movement, confinement, and spiritual advancement from a literary‐narrative point of view, thus deepening our understanding of asceticism and monasticism in Byzantine contexts.

Hagiography is the most prolific genre of Byzantine literature and offers modern scholars insights into the Byzantine understanding of the concept of holiness. 1 All hagiographic subgenres, including the *Lives* of ascetics and monastics, which were particularly popular among the Byzantines, endeavour to sketch the holiness of their heroes and heroines in vivid detail. 2 Wanderings outside monastic walls as well as self‐confinement within monasteries and other enclosed or secluded spaces seem to be integral components of such stories of holiness. This tripartite relationship between movement, confinement, and sanctity has hitherto never been examined by modern scholarship from a literary vantage point. 3 By analysing text examples dating to the early and middle Byzantine periods, this contribution shows how ascetics and monastics are depicted in hagiographical narratives as ascending to the state of holiness through both their mobility and their self‐imposed confinement.

As will be demonstrated below, whether they are in populated settlements or in the desert, holy men and women seek isolation in confined or secluded places, so much so that they sometimes feel compelled to wander from place to place to achieve this goal. More specifically, self‐confinement (ἐγκλεισμός), the practice of leading a non‐worldly – often solitary – way of life in an enclosed place, is the result of ascetics’ and monastics’ longing for isolation; their desire to be far away from mundane people, or any other humans at all, 4 can lead them to travel long distances until they find or build their enclosed retreat. In ascetic contexts, isolation is thus the motivation that drives holy men and women to confine themselves in a remote place, and mobility is the means to approach that place in which their spiritual transformation will be completed.

Athanasios’ *Life of St Antony of Egypt* (*BHG* 140/*CPG* 2101), 5 which was written in the fourth century and is considered to be the most ancient Greek *Life*, constitutes an inevitable starting point for the present study. Antony resorts to enclosed and secluded spaces in order to intensify his ascetic practices, first in a grave at a cemetery outside of town and then in a derelict abode inside a ruined fort located in the desert. According to the text, his stay in the deserted building was of fundamental importance for his spiritual progression: ‘Antony emerged as though from some shrine, having been initiated into divine mysteries and inspired by God’. 6 Then Antony appears as a leader of a monastic community in the desert, and the story reaches its climax when he goes to live as a hermit in an absolutely isolated space, namely ‘the interior desert’. 7 All of his movements were evidently aimed at achieving isolation and spiritual perfection by living in seclusion from the world whether inside a monastery or in solitude in the desert.

Antony’s ascetic and monastic lifestyle exerted considerable pull on the way later ascetics and monastics led their lives in imitation of his example, and his *Life* influenced later hagiographers, who strove to present the lives of their own protagonists in a way that evokes an association with the paradigm of Antony. This applies not only to most cases of male ascetics and monastics but also to a few of their female counterparts. 8 In what follows, I will discuss two selected *Lives* of female saints from the early and middle Byzantine periods that offer a vivid picture of the connection between movement, confinement, and spiritual advancement: the *Life of St Mary of Egypt* (*BHG* 1042) and the *Life of St Matrona of Perge* (*BHG* 1221 and 1222). These two texts yield insights into intriguing aspects of (fe)male sanctity achieved through the combination of wanderings and confinement by focusing on both female *and* male asceticism *and* monasticism. In the stories in question, the female protagonists are constantly on the move in their search for isolation, which is an extremely unusual element in the *Lives* of female saints: more often than not, such texts depict nuns enclosed in their houses or convents, following the female monastic model provided by the fourth‐century *Life of St Makrina* (*BHG* 1012). 9 It is notable that male monastics also play a crucial role in the plot lines alongside the female protagonists (Mary of Egypt; Matrona of Perge), who are largely described as having male (or non‐female) characteristics.

Scenes of mobility and confinement in the context of asceticism and monasticism, as described in the selected texts, reveal an image of holiness which, albeit unusual, would have been widely known among the Byzantines. The seventh‐century *Life of St Mary of Egypt* (*BHG* 1042) was incorporated in the exceptionally popular tenth‐century *Menologion* of Symeon Metaphrastes without alterations, 10 while the earliest known version of the *Life of St Matrona of Perge* (*BHG* 1221), written in the mid‐sixth century or later, was also included in Symeon’s *Menologion*, yet in a thematically and stylistically revised version (*BHG* 1222). 11 The stories of Mary of Egypt and Matrona of Perge will be examined here through a literary‐critical analysis of all their versions mentioned above. Focus will be placed not only on the relevant scenes which remained unchanged throughout several centuries, but also on those scenes which were subjected to variations by Symeon Metaphrastes in the tenth century, as both types of literary representations can broaden our understanding of the spectrum of Byzantine conceptions of asceticism and monasticism.

## Seeking isolation in the desert: the *Life of St Mary of Egypt* (*BHG* 1042)

The *Life of Mary of Egypt* is the text *par excellence* in which the desert exemplifies a secluded place that decisively facilitates isolation and spiritual progression within the framework of an ideal ascetic way of life. In fact, practices of solitary and cenobitic life, as presented in this text, are connected with and benefit from the environment of the desert, which, despite its geographic openness, serves as a place of ascetic confinement. Monasticism and asceticism face each other in the encounter of the story’s two protagonists: Zosimas is a Palaestinian monk who abandoned his initial monastic community to live in a monastery situated in the vicinity of the river Jordan bordering the desert. On one occasion, Zosimas leaves this monastery and walks deep into the desert, where he meets Mary, a repentant prostitute who had left Alexandria to come to Jerusalem whence she departs to lead a solitary life in the desert for forty‐seven years. Mary tells the story of her life to Zosimas, who visits her two more times over the next two years: first, to bring her the holy communion and again to bury her dead body in the desert. Zosimas subsequently makes her life known and dies as a monk much later at almost one hundred years of age. The text begins and ends with the story of Zosimas, while the story of Mary is embedded in the middle. 12 Both protagonists seem to benefit from the other’s presence and their interaction along their respective paths to holiness. Ironically, it is their wanderings and common wish to achieve isolation that is the impetus leading to their encounter in the desert. As will be shown below, wanderings and self‐confinement in the desert are key elements in the *Life of Mary* determining both the lives of the two protagonists and the narrative structure of the text.

At the very outset, the anonymous hagiographer of the *Life of Mary* points to the significance of movement in achieving isolation as an essential aspect of spiritual advancement. Even though Zosimas was an accomplished monk engaging in every kind of abstinence and ascesis, at some point he committed the sin of pride by vexing himself with the thought of having acquired all possible knowledge and having attained perfection in every way. 13 A divine voice immediately prompted him to leave the monastery in his hometown where he had been practising asceticism since childhood and to retreat to a monastery near the Jordan River at the edge of the desert, ‘In order […] to learn how many other ways lead to salvation.’ 14 This command underscores the idea that seeking even greater isolation in monastic confinement, far removed from a familiar environment, could contribute significantly to moral and spiritual progression. 15 Indeed, by observing the lifestyle of the other monks in this remote monastery near the river Jordan, especially the fact that they participated in all‐night vigils and consumed only bread and water for sustenance, Zosimas also felt disposed to intensify his own efforts and spiritual struggles. 16

The significance of confinement and isolation is also stressed by the hagiographer himself, who thoroughly describes the exact location of Zosimas’ new monastic dwelling, which actually constituted a physical border (or rather, a liminal space) between the city and the desert: the monastery had no contact with laypeople, and it was located in such a rugged and isolated place that even most monks from other monasteries were unable to find their way to it. As such, this monastery appears to be a special place of confinement and isolation that few could enter. At this point, the text states:

[Normally] the gate of the monastery was never opened but remained always shut, providing in this way the opportunity for the monks to pursue their ascetic life undisturbed. In fact, it was not permitted for the gate to be opened, except perhaps when a monk would come [there] in great need. For it [i.e., the monastery] was [in] a secluded location, and it was not only inaccessible, but also unknown to the majority of the neighbouring monks. 17

Because of Zosimas’ sincere urge and strong need to grow spiritually, he was allowed to stay in the monastery.

One gets the impression that the hagiographer is consciously and systematically seeking a balance between scenes of confinement in the monastery and scenes of wanderings, since, right after the description of the monastery’s isolated location and Zosimas’ admittance to it, the text immediately turns to a special rule of the monastery that helps push the story to its climax: in order to intensify their ascesis and contemplation, once a year the monks left the monastery to spend the forty‐day period of Lent alone in the desert. According to the text, ‘As soon as they crossed the [river] Jordan, they separated and moved far away from each other and’, like the monks portrayed in the *Life of Antony*, ‘made the desert their city’. 18 Subsequently, the anonymous hagiographer again emphasizes that even during their wanderings in the desert, the monks should live separately and avoid contact with each other in order to facilitate their communication with God. In this sense, the amalgamation of wanderings and self‐confinement in the desert proves to be of crucial importance, or even a prerequisite, for the spiritual connection with the divine, especially during Lent, which was a time of intensified spiritual life in imitation of Christ’s forty days in the desert (cf. Matthew IV.1–11; Mark I.12–13; Luke IV.1–13).

The hagiographer does not fail to underline that, whilst secluded in the desert, Zosimas was constantly on the move, ‘never relaxing the pace of his movement’. 19 He wished to enter ‘the innermost part of the desert’ as soon as possible, in the hope that he would meet a holy father there to teach him. 20 Indeed, after twenty days of wandering, he finally saw from afar a figure with relatively short and sparse white hair and with a naked body darkened from exposure to the sun. He wanted to learn who *this man* (ὁ ὁρώμενος) or *this creature* (τὸ ὁρώμενον) was. 21 Obviously, Zosimas was unable to recognize the female identity of Mary as she had lost all female bodily characteristics, especially her long full hair and her breasts. Zosimas starts chasing this figure, and only after an exhausting run does Mary disclose that she is a woman and ask him to throw her his cloak to cover her naked body before moving closer. By enveloping herself in Zosimas’ cloak, Mary seemingly assumes the identity of a monk, which is, in fact, an additional sign of her non‐female, or rather – in Zosimas’ perception – her male, identity. 22 Zosimas is also impressed that Mary already knows his name and his rank as a priest, and so he is convinced of her holiness and sees in her the opportunity to achieve the initial goal for which he left the monastery and engaged in this long wandering in the desert, namely, to familiarize himself with other kinds of asceticism and thereby enhance his spiritual wisdom. 23 In fact, he expects her to function as the intercessor between himself and God. He thus implores her to pray for him and to recount in detail the story of her previous life for edifying reasons.

In the course of her story, we can observe that Mary was a woman who was incessantly on the move, first as a prostitute in a city and then as an ascetic in the desert. Already at the age of twelve, the Christian heroine abandoned her family and went to Alexandria where for more than seventeen years she led a dissolute life having sexual intercourse with men not out of financial need but for pure pleasure. One day, she approached the sea and saw there a host of men preparing to go on pilgrimage to Jerusalem. She travelled with some of them, whom she also seduced on board the ship on the way to the Holy City. Despite her sinful behaviour on the ship, the sea voyage from Alexandria to Jerusalem represents a crucial point in her life that would eventually change her conduct from sinful and dissolute to spiritual and holy. In this context, the sea becomes the means of escape from a sinful life and turns out to be a symbol of the change that would follow. Mary herself even wonders in hindsight about the ‘tolerance’ of the sea in allowing her to travel safely despite her sins, but in the end, she attributes her safe passage to divine providence: ‘I am truly surprised […] how the sea endured my profligacy […]! But, as it seemed, God sought my repentance.’ 24 Indeed, in Byzantine hagiography, the sea is often used metaphorically to denote the hardships and temptations that may emerge in one’s life. 25 In the case of Mary, the infinite sea is paralleled by the infinite sin she committed as a prostitute. Yet by crossing the sea, she comes closer to annihilating her sinful life as God wills. In other words, this journey has been designed by God to lead her to penitence and holiness.

Upon arriving in Jerusalem, she spent some time as a prostitute, and this sojourn too was a critical step in her transformation. On the feast day of the Exaltation of the Holy Cross, Mary tried in vain to enter a church, perhaps that of Constantine at Golgotha. 26 This experience helped her realize that she should start leading a life in penitence. By the agency of the Virgin Mary, she managed to visit the church and pay reverence to the wood of the Holy Cross, while a divine voice impelled her to retreat to the desert by crossing the Jordan River. The following scene, connected to her conversion and withdrawal into the desert, is also described as one continuous movement: walking through the city and asking for directions, she finally arrived at the church of John the Baptist, prayed there, and then proceeded to the river Jordan where she washed her face and hands. This action could be seen as a symbolic baptism in imitation of Christ’s baptism in the Jordan, signifying her change of heart and her new identity as a repentant prostitute and as an ascetic. 27 She then returned to the church of John the Baptist to receive the holy communion. The next day, she crossed the river in a small boat to enter the desert, where Zosimas found her forty‐seven years later. 28

As mentioned above, during the first stage of her life, Mary was constantly engaged in movement and travels whilst seeking lovers and sexual intercourse, which are activities that exceeded the limits of a woman in the patriarchal Christian society of Byzantium from both a religious and a social point of view: a Byzantine woman was expected to have been a virgin or a conventional housewife, avoiding a public social life. 29 As a prostitute and a constant traveller within and beyond the borders of her city, Mary represents the opposite of this ideal. Not only during her sinful life in the city, but also during her sojourn in the solitude of the desert, she seems to be in a state of endless wandering, fighting with memories of her past and seeking repentance. According to the text, Mary’s mobility throughout her sinful life lasted about seventeen years – a period that corresponds to the number of years she needed to fight off the severe challenges and temptations in the desert, before spending another thirty years there and finally encountering Zosimas: ‘for seventeen years I wandered in this desert struggling with those irrational desires, as if with wild beasts’. 30 In this manner, the hagiographer draws a parallel between Mary’s life as a sinful woman and her life as a repentant sinner. It seems that Mary actually strove to ‘replace’ the previous form of her (sinful) mobility with a new one connected to her repentance and asceticism. To put it another way, her (ascetic) wandering became the means through which, in the end, she overcame her wayward past and achieved holiness.

And it is exactly this, her constant mobility, that distinguishes this female protagonist from other holy women: Mary of Egypt, along with her quasi‐replica figure Theoktiste of Lesbos, 31 is the only example of a female solitary living and wandering in the wilderness for such a long period. In this context, Mary’s physical transformation from female to almost male can be further elucidated. Apart from the male or non‐female characteristics of her body discussed above, she resembles men also in her way of asceticism, especially her constant wandering in the desert, which is often attributed to male ascetics such as Antony the Great and Paul of Thebes, who lived and wandered in the desert for the better part of their existence. 32 Thus, in my opinion, Mary’s constant wandering, narratively and iconographically visualized in her emaciated body and her sun‐scorched skin, 33 proves to be a further characteristic of her masculine self, which a woman had to achieve in one way or another in order to attain holiness. 34

The impression that Mary, although living in the open and unwelcoming desert for forty‐seven years, never ceased to be on the move is reinforced by the fact that the hagiographer does not mention any dwelling place such as a cave, in which she could have found refuge from the searing heat or other hazardous weather conditions. One can argue that the hagiographer deliberately wished to create this impression, especially if one considers that the earliest version of this *Life*, a short account included in the *Life of St Kyriakos* (*BHG* 463; *CPG* 7538), written by Cyril of Skythopolis in the sixth century, 35 refers to a cave as Mary’s hermitage in the Judean desert. Consequently, the hagiographer of Mary’s *Life*, who presumably knew this brief version and even drew on it, 36 tacitly, yet ingeniously, removed this important detail from the story. In this manner, the author distinguishes his female protagonist from both male ascetic paradigms of Antony and Paul of Thebes, who, living in caves or other hermitages, were not completely exposed to the hardships of the desert. Thus, Mary’s extreme asceticism and, by extension, her masculine identity surpasses that of these male ascetic figures. In an attempt to accent the unique holiness of his heroine, the hagiographer of the *Life of Mary* actually overshadows the importance of the experience of Antony’s enclosure in the derelict fortress and subsequent spiritual benefits by presenting his own heroine as being overexposed to all dangers of the desert, through which she eventually gained her spiritual perfection. Moreover, Mary appears to be superior to Antony for an additional reason: unlike Antony, 37 Mary enters the desert and never abandons it, making her an ideal ascetic paradigm. Therefore, for Mary, the desert serves as a kind of enclosed space, in which she chooses to remain confined and spend the rest of her life in repentance. As she says, ‘I came to this desert, and since then to this day *I have fled afar off and lodged in this [wilderness], waiting for* my God.’ 38 In this paradoxical way, Mary is simultaneously both confined and on the move. Her mobility proves to be not only the means to approach the desert, which is her final destination and destiny, but also a modus of ascesis within this place.

As we have seen, both protagonists of this text, Zosimas and Mary, experience a kind of confinement and movement as part of their ascetic way of life. Although at the outset the life of Zosimas and that of Mary are diametrically opposed to each other (Zosimas is a monk while Mary is a prostitute), at some point, in fact, their lives seem to converge as regards their movement, confinement, and spiritual advancement. More specifically, the reason behind the need to abandon their hometown and embark on a long journey to Jerusalem is their moral transgression: a divine voice helps Zosimas realize the sin of pride he has committed and urges him to seek moral improvement and salvation in another monastery. And Mary, even though she is not aware of the importance of her travels at the time, interprets it retrospectively as the result of God’s providence, who wished to return her to the right path. During her stay in Jerusalem, she also realizes her lapse and hears a divine voice, whose instructions she, like Zosimas, follows to the letter. In this way, both protagonists find themselves ‘confined’ in the desert area outside of Jerusalem, pursuing an ascetic lifestyle: Zosimas in a monastery on the outskirts of the city and close to the desert, and Mary in the desert’s very heart. It is here that the two protagonists finally meet. However, some essential differences in the otherwise parallel course of their lives can also be observed: Mary enters the desert seeking repentance, whereas Zosimas is seeking to be humbled by someone spiritually superior. In the end, through their experiences and hardships in the desert, both protagonists grow spiritually and come ever closer to salvation and holiness. 39 Yet, unlike Zosimas, who after their encounter leaves to return to his monastery, i.e., his place of confinement, Mary remains in the desert, which operates as her own ascetic place of self‐confinement, and continues her wandering there until Zosimas finds her dead body and buries it with the help of a lion.

Finally, Zosimas shares Mary’s story with his fellow monks and makes her known as the saint who incessantly wandered in the solitude of the desert in quest for repentance. 40 Thus, although she dies physically and her constant wandering in the wilderness ceases, her story is left behind and continues to circulate, both in oral and written form, thereby presenting and preserving Mary’s perpetual movement not only in monastic circles but also among laypeople. In addition, Symeon Metaphrastes included this story unaltered in his tenth‐century liturgical collection, further cementing its popularity. The *Life of Mary of Egypt* challenged (and still challenges) the way Christians are expected to perceive its female protagonist: although her conversion from a prostitute to a repentant sinner gives hope of salvation to every Christian, 41 the depiction of Mary as an extreme ascetic figure actually offered (and offers) an example to admire rather than to imitate. 42 And this applies not only to laypeople but also to ascetics and monastics. Nobody matched her way of ascesis.

## Seeking isolation in the city: the *Life of St Matrona of Perge* (*BHG* 1221 and 1222)

In his prologue, the anonymous pre‐Metaphrastic hagiographer points out that this is ‘the *Life*, full of benefit for our souls, of the blessed and holy Matrona, a woman who […] displayed the traits of holy men in the midst of monastic men and mastered the feats of accomplished desert solitaries’. 43 Symeon Metaphrastes’ tenth‐century version accentuates Matrona’s spiritual achievements by clarifying that ‘She competed with men in virtue and surpassed them in marvelous accomplishment.’ 44 Matrona incorporates manliness not only because she belongs to the category of holy women who denied their female identity by dressing in men’s clothing and entering a male monastery, 45 but also because, like Mary of Egypt, she is distinguished by her constant mobility, which is a common characteristic of male ascetics and monastics. In fact, in the course of their lives, almost all women monastics who assume male garb engage in some short‐ or long‐distance travels in an attempt to escape notice and keep their female identity secret. However, Matrona’s travelling or wandering from one city to another is so extensive that it constitutes a particularity of her *Life*. Earlier scholars of the *Life of Matrona*, such as Hippolyte Delehaye, Cyril Mango, and more recently Stavroula Constantinou, have already pointed out this distinctive feature of the text, 46 yet without offering any interpretation of its function in the development of the plot.

Each sequence of Matrona’s movements ends up in self‐confinement. This recurring motif pervades the structure of the whole text. And it is exactly because of this combination of movements and confinements that this *Life* ‘is distinguished by its length, [which is] unusual for a female saint’, 47 as Mango rightly observed. In the following, the analysis will focus on two parts of the story that start with travelling and end with confinement in a city, during which the female protagonist was on a quest for isolation and spiritual progression. First, along with her husband Dometianos and her daughter Theodote, Matrona leaves her hometown of Perge to settle in Constantinople, where she hopes to lead a more religious life. However, her abusive husband raises objections to this way of life and tries to restrain her. Soon thereafter, she abandons her family (her daughter dies in the meantime) to enter a male monastery, whose abbot was a monk called Bassianos. Here she lives for three years disguised as a eunuch called Babylas. In this way, the first series of movements is completed with Matrona having achieved her goal to become a monastic. Then, Matrona’s whereabouts are discovered by her husband, who proceeds to chase her down, resulting in a second series of movements and travels to various cities which lead the text to its climax. Matrona eventually finds refuge in a deserted pagan temple located in a secluded area of the city of Beirut. She lives there as an ascetic for a long time and gradually gathers a small community of solitary women. Finally, she returns to Constantinople, where she expands her female community and eventually founds her own monastery with the help of Bassianos and his assistant Markellos. Matrona dies as the abbess of the monastery at an old age, recognized as a saint because of her overall ascetic and monastic career. As can be seen from this summary, the whole story revolves around two pivots – mobility and confinement, which go hand in hand with the spiritual advancement of the female protagonist.

Both movements included in the first part of the story, namely the journey from her hometown in Anatolia to Constantinople and later from her new home there to Bassianos’ monastery, are described in both versions examined here as the result of God’s providence. 48 From the very first moment in Constantinople, Matrona leads a pious life by visiting sanctuaries, fasting, and praying for days and nights on end. Despite the prohibitions of her husband, she keeps attending the church services, whereupon she dreams that she is pursued by her husband and eventually rescued by monks. Matrona interprets this dream as a sign that she should enter a male monastery, and this is how ‘God’s grace’ brings her to Bassianos’ monastery. 49 For this purpose, she takes on the appearance of a eunuch by assuming male attire and a man’s haircut. Through her intensive spiritual and ascetic practices, Matrona, who passes as the eunuch Babylas, manages to gain the admiration of her male fellows and even to become an example they strive to imitate. 50 When her female identity is finally revealed to Bassianos in a dream, Matrona admits that she arrived at the monastery by following ‘God’s guidance’. 51 This is a key concept: God appears to function here as a guide (*Wegweiser*) in a topographical sense, as it is God who shows Matrona the way to Bassianos’ monastery and ensures that she reaches her destination.

Both versions of the text create the impression that Matrona’s actions, including all her movements and her decision to enclose herself in a male monastic community, are God‐pleasing. This authorial strategy has a twofold aim: first, to justify the decision of the heroine to assume men’s clothing and enter a male monastery, although such behaviour had been prohibited by the official church; 52 second, to present her movements and confinements as crucial points in her ascetic career, which by the grace of God would ultimately lead her to spiritual perfection and holiness. Obviously, after the three‐year sojourn and hard spiritual practices among men in the monastic community, God saw that she was ready to further intensify her ascesis by moving to another location where she could be fully isolated. This is the reason why Bassianos was informed of her true identity by divine intervention.

However, Matrona incorporates her manliness to such an extent that she is unable, at that moment, to see into God’s ultimate plan for her and literally laments when she is asked by Bassianos to abandon the monastery:

She moaned, saying, ‘Woe is me, wretch that I am! For I am cast out as one unworthy. Woe is me, miserable one that I am! For it has been discovered what I am, and I am no longer counted a brother among the brethren; no longer am I thought to be a eunuch, nor to be addressed as Babylas, but am soon once again to be a woman and to be called Matrona’. 53

According to the pre‐Metaphrastic passage above, Matrona regards the revelation of her true sex and the subsequent renunciation of her male self as complete destruction of all her efforts. Τhe heroine has identified so strongly with her role as a male monk through her recent behaviour and appearance that she does not want to return to her initial gender identity. By contrast, Symeon Metaphrastes, who already in his prologue stressed the exemplary ascetic behaviour of his female protagonist, must have omitted this extreme emotional outburst from his account intentionally. 54 By doing so, he may have wished to indicate that Matrona’s transition from a eunuch and male monk back to her initial gender identity was completed smoothly and in accordance with her own full consent to Bassianos’ request. Apparently, Symeon Metaphrastes aimed to avoid depicting his heroine as a weak woman or as a woman completely powerless in the face of a man’s decisions. He thus trivializes the chasm that separates Matrona’s self‐assumed identity of superior ascetic masculinity, which she had worked hard to achieve, from the female identity that the male abbot demands she return to. Under these circumstances, she abandons the male monastery and at the same time her male identity. Now recognizable again as Matrona, the only way to protect herself and her spiritual lifestyle is by substituting the shift in her gender (to which Bassianos had put an end) with a deliberate shift in location, moving away from Constantinople where her husband is. Thus, the story proceeds to Matrona’s second sequence of movements and confinements.

Bassianos approves Markellos’ proposal to send her by ship to a convent in the city of Emesa. 55 Like Mary of Egypt’s hagiographer, the pre‐Metaphrastic author stresses here that the successful completion of Matrona’s ship journey should be attributed to God, who supported her throughout her spiritual pursuits. 56 In Emesa, Matrona lived as a nun in a convent for a long time and eventually became an abbess, yet both versions of the text present her confinement experience there very briefly. When located by her husband Dometianos, Matrona abandons Emesa, too. Then three travels succeed each other without any long sojourn in between. First, Matrona sets off for Jerusalem. Upon her arrival there, she intensifies her spiritual struggles by staying overnight in various churches of the city. Soon thereafter, she is informed that her husband is looking for her in the Holy City, and she leaks information about her alleged plan to go to Paneas (Caesarea Philippi, north of Jerusalem), whereas she actually journeys to Mt Sinai, in the opposite direction. 57 The significance of movements in the narrative is underscored through the actions of Matrona herself, who, beyond ‘actual travelling’, now uses ‘false travelling’ in an effort to confuse and escape from her husband. But Dometianos learns of her real plans and follows her to Mt Sinai. Without any delay, Matrona abandons Mt Sinai and goes to Beirut in Lebanon. Overall, leaving Constantinople and Emesa behind, Matrona continues travelling around the Byzantine Near East, which can be paralleled by the constant movement of Mary of Egypt, who never ceases her wanderings in the innermost parts of the desert as a form of ever harder ascesis. Still, although Matrona repeatedly changes her city of residence, she remains in an urban environment or in its immediate vicinity.

In Beirut, Matrona chooses to dwell in a desolate and secluded pagan temple, where she lives in ascetic confinement for many years. The temple must have been on the outskirts of the city or not very far from the city’s limits. Both the pre‐Metaphrastic and Metaphrastic versions underline the importance of this ascetic experience in the spiritual development of the protagonist by thoroughly describing the relevant events. However, the pre‐Metaphrastic version contains details that the Metaphrastic version does not – specifically details about the place and the challenges the heroine is there called on to face:

Now, it happened once, as she performed the nightly psalmody, that demons sang most fervently in response, for she heard the voices of many men singing. Taking fright and fortifying herself with the sign of the cross, she completed the psalmody, considering within herself and saying: ‘this place is deserted and the house unhallowed; there is no populated place in this area, nor have any passers‐by approached; whence, then, come these voices?’ 58

Matrona’s thoughts are exposed to give a description of the place and to introduce the attack of the demons. According to late antique belief, the desert itself or other deserted places (in this case, the environs of the city, including the pagan temple) are inhabited by pagan gods who act in the form of demons, and by entering such places, ascetics inevitably come into an outright confrontation with the local evil spirits. 59 As such, Matrona begins leading an ascetic way of life similar to that of Antony, who had to face the demons first in a grave and then in a deserted fortress, before finally gaining control over them. 60 Both Matrona and demons fight for supremacy over their immediate surroundings, and, like Antony, Matrona manages – despite the sustained attacks of the demons – to fend them off.

Matrona’s recent place of confinement is depicted with two contradictory facets which underscore its crucial importance in her spiritual formation. On the one hand, the place appears to actively contribute to her ascetic struggles by providing her with what she needs for subsistence: ‘Thus did that place supply her ever after, as if by way of tribute, with the amplest daily nourishment.’ 61 On the other hand, a demon that appeared in the form of a woman reminds Matrona how unwelcoming and dangerous this place is: ‘This is no place for you: it is a dwelling of idols and demons. Come to Beirut [i.e., the inner city], for it is a beautiful and hospitable city, which provides for all people. It is desolate here: there is no provision for the necessities of life.’ 62 The demons endeavour, in vain, to send her back to the inner city in order to hinder her spiritual progress and thereby regain their territory. The demons’ effort and, at the same time, Matrona’s persistence in staying in the area demonstrate that confinement in an isolated place is essential for asceticism and the pursuit of holiness, also within an urban environment.

Matrona’s constant movements, albeit externally imposed, serve in her life as forms of ascetic practice which, in conjunction with the experiences of self‐confinement, help the heroine reach a higher level of spirituality. One of these aspects on its own would not be sufficient for achieving holiness. 63 This function of mobility and confinement can be best understood by considering the end of the story: after all her travels and periods of confinement described above, Matrona proceeds with one last journey to return to and remain in the place where everything began, thereby fulfilling God’s purpose for her life. 64 Matrona crosses the sea and moves back to Constantinople, no longer having anything to fear from her husband. In fact, after her resettlement in the Byzantine capital, neither of the two versions refers to Dometianos or the heroine’s thoughts about him, which indicates that something has changed between the outset and the end of the story. Matrona has grown spiritually so much that she even establishes her own monastic community, first on a smaller scale in Beirut, and then an extensive one in Constantinople with the aid of Bassianos and Markellos. Symeon Metaphrastes describes the precise location in Constantinople where Matrona’s convent was built: ‘[the place] had the sea on the right side, and on the other, it neighboured the monastery of Bassianos’. 65 It is highly interesting that in the end, Matrona finds herself located between the two elements that came to determine her identity and her reputation: her first place of confinement (Bassianos’ monastery), where she began her monastic career as a monk in male attire; and the sea (her first and final mode of transportation), symbolizing her intense activity of moving from one place to another.

In addition, according to the pre‐Metaphrastic version, Bassianos allows Matrona and her nuns to wear male attire in their everyday lives, 66 meaning that Matrona’s previously unique adoption of men’s clothing now becomes an element of the identity of her entire monastic community. Symeon Metaphrastes completely omits this information, either because this mode of dress was not practised during his time, 67 or because he wished to honour Matrona as a predominantly *female* holy figure. In the Metaphrastic version, she is no longer presented as wearing men’s clothes and Bassianos’ permission to do so is thus redundant. In this manner, Symeon Metaphrastes indirectly, yet clearly, demonstrates that Matrona’s period of dressing as a man was just one of several previous stages of life that ultimately helped her achieve advanced spirituality *as a woman*, and at the same time affirms the heroine’s gender identity, removing any possibility for confusion in this regard at the closure of the text. Furthermore, although the pre‐Metaphrastic version repeatedly mentions that Matrona was trying to imitate Bassianos in all matters, 68 the Metaphrastic version concludes by leaving Bassianos’ role aside to underscore once again Matrona’s own agency and special achievements, which, like those of Mary of Egypt, were to be admired rather than imitated: ‘Then she left this world to her beloved Christ, having left behind her actions that were widely admired, but capable of being imitated by only a few.’ 69 Indeed, her constant mobility from one city to another in quest of isolation makes her ascetic career unique. First as a monk and then as a nun, solitary, and abbess, Matrona incorporates all aspects of asceticism and monasticism, both male and female.

## Conclusions

This contribution aimed to show that stories of holiness, though widely discussed in modern scholarship, still have a great deal to offer if one sheds light on their scenes of wanderings and confinements. Both texts examined here, the *Lives* of Mary of Egypt and Matrona of Perge dating from the early Byzantine period, recount stories whose male and female protagonists (Mary and Zosimas; Matrona), whether in towns or in the desert, feel compelled to abandon their initial place of residence to find isolation and practise asceticism in confined or secluded places. In this context, their mobility – within the urban environment or in the wilderness – appears to be a prerequisite for their spiritual ascent. The much sought‐after isolation achieved through confinement serves as a metaphorical death and rebirth, as the protagonists leave their worldly or sinful life behind to lead a holy life for Christ’s sake.

The notion of movement is also employed in these two narratives as a rhetorical device for their development. Both stories follow a cyclical style of narration, meaning that the authors bring their story back to the starting point after recounting all the journeys and hardships that their protagonists experience. Specifically, the *Life of Mary of Egypt* uses Zosimas’ monastic career and its vicissitudes as a frame story within which Mary’s story is nested, while the *Life of Matrona*, and in particular her spiritual path, begins and ends with the heroine’s monastic confinement in Constantinople. At the outset, Matrona, disguised as a monk, encloses herself at Bassianos’ monastery in Constantinople. Many years later and after a series of travels, she returns to Constantinople and, next to the monastery of Bassianos, establishes her own convent, in which men’s dress is worn by the nuns. Thus, the narrative closely follows the extended journey that Matrona experiences and even imitates it in its circular movement.

Finally, these two stories propose different conceptions of monasticism and asceticism, based on the particularities of each protagonist’s way of life. Despite their differences, they exhibit a substantial similarity, namely the depiction of the protagonists’ spiritual advancement achieved through movements and confinements. In other words, Matrona’s travels within an urban environment are comparable to Mary’s wanderings in the desert.

In the tenth century, Symeon Metaphrastes integrated the *Life of Mary of Egypt* into his liturgical collection without alterations, whereas he chose to revise the *Life of Matrona of Perge* stylistically and thematically to accentuate the role of a female figure in the – otherwise male‐dominated – monastic and ascetic community of Byzantium. In both cases, Symeon Metaphrastes maintained the two components that lead the protagonists to holiness: mobility and confinement. Like Mary of Egypt and Matrona, Zosimas also achieved spiritual perfection through monastic confinement and wanderings. In sum, the examined texts, in all their versions, attest that no matter how advanced one is in worldly and spiritual wisdom, through the combined experience of moving and being enclosed in remote and secluded places, one can progress further along the path of corporeal and spiritual ascesis.

## Footnotes

^1^
Hagiographical texts, more than any other literary genre from Byzantium, are preserved in a plethora of manuscripts, see A. Ehrhard, *Überlieferung und Bestand der hagiographischen und homiletischen Literatur der griechischen Kirche von den Anfängen bis zum Ende des 16. Jahrhunderts*, Texte und Untersuchungen zur Geschichte der altchristlichen Literatur 50–52.2, 3 vols (Leipzig, 1936–52).

^2^
M. Hinterberger, ‘Byzantine Hagiography and Its Literary Genres. Some Critical Observations’, in S. Efthymiadis (ed.), *The Ashgate Research Companion to Byzantine Hagiography*, vol. 2: *Genres and Contexts*, Ashgate Research Companions (Farnham and Burlington, 2014), pp. 25–60, esp. at p. 28.

^3^
Here it is worth mentioning a recent study that touches upon literary aspects of mobility, confinement, and sanctity in regard to early Christian martyrs, see C. Papavarnavas, ‘Imprisoned Martyrs on the Move: Reading Holiness in Byzantine Martyrdom Accounts’, *Byzantinische Zeitschrift* 114.3 (2021), pp. 1241–62; open access: https://www.degruyter.com/document/doi/10.1515/bz‐2021‐0061/html?lang=en. Specifically on the confinement of holy martyrs, see C. Papavarnavas, *Gefängnis als Schwellenraum in der byzantinischen Hagiographie: Eine Untersuchung früh‐ und mittelbyzantinischer Märtyrerakten*, Millennium‐Studien 90 (Berlin and Boston, 2021); open access: https://www.degruyter.com/document/isbn/9783110725780/html. For an overview of the importance of mobility in hagiographical texts, see S. Efthymiadis, ‘Νοεροί και πραγματικοί ταξιδιώτες στο Βυζάντιο του 8ου, 9ου και 10ου αιώνα’, *Byzantina* 20 (1999), pp. 155–65 (with further references).

^4^
For different levels of isolation in ascetic contexts, see A.‐M. Talbot, *Varieties of Monastic Experience in Byzantium, 800–1453*, The Conway Lectures in Medieval Studies, 2014 (Notre Dame, 2019), esp. pp. 111–13.

^5^
F. Halkin, *Bibliotheca Hagiographica Graeca*, Subsidia Hagiographica 8a, 3rd edn, 3 vols (Brussels, 1957) [hereafter *BHG*]; M. Geerard (ed.), *Clavis Patrum Graecorum*, 5 vols, supplementum (Turnhout, 1974–98) [hereafter *CPG*].

^6^
Athanasios of Alexandria, *Life of Antony*, c. 14.2, ed. G.J.M. Bartelink, *Athanase d’Alexandrie, Vie d’Antoine: introduction, texte critique, traduction, notes et index,* Sources Chrétiennes 400 *(Paris, 1994),* pp. 124–377, at p. 172: προῆλθεν ὁ Ἀντώνιος ὥσπερ ἔκ τινος ἀδύτου μεμυσταγωγημένος καὶ θεοφορούμενος. English trans. T. Vivian and A.N. Athanassakis, *Athanasius of Alexandria, The Life of Antony: The Coptic Life and The Greek Life*, Cistercian Studies 202 (Kalamazoo, 2003), pp. 51–259, at p. 91.

^7^
Athanasios of Alexandria, *Life of Antony*, c. 49.4, ed. Bartelink, p. 268: εἰς τὴν ἐσωτέραν [or ἐνδοτέραν] ἔρημον. English trans. Vivian and Athanassakis, *Athanasius of Alexandria, The Life of Antony*, p. 165.

^8^
For the idea that female ascetics and monastics could also have male saints as their role models, see C. Rapp, ‘Figures of Female Sanctity: Byzantine Edifying Manuscripts and Their Audience’, Dumbarton Oaks Papers 50 (1996), pp. 313–43, esp. at pp. 314, 326–9.

^9^
Gregory of Nyssa, *Life of Makrina*, cc. 5–7, 11, ed. P. Maraval, *Grégoire de Nysse, Vie de Sainte Macrine: introduction, texte critique, traduction, notes et index,* Sources Chrétiennes 178 *(Paris,* 1971), pp. 136–267, at pp. 154–64, 174–80. Cf. S. Constantinou, *Female Corporeal Performances: Reading the Body in Byzantine Passions and Lives of Holy Women*, Studia Byzantina Upsaliensia 9 (Uppsala, 2005), pp. 127–8.

^10^
C. Høgel, *Symeon Metaphrastes: Rewriting and Canonization* (Copenhagen, 2002), esp. at pp. 92 n. 16, 201. For the edition of the *Life of St Mary of Egypt* (*BHG* 1042), see *Patrologia Graeca* 87, cols 3697–726. This text was presumably written in the seventh century, and the Byzantines erroneously attributed it to Sophronios, Patriarch of Jerusalem (634–8). For a discussion of the date of composition and Sophronios’ authorship of the text, see M. Kouli, ‘Life of St. Mary of Egypt’, in A.‐M.Talbot (ed.), *Holy Women of Byzantium: Ten Saints’ Lives in English Translation*, Byzantine Saints’ Lives in Translation 1 (Washington, DC, 1996), pp. 65–93, at p. 66 (with further references); B. Flusin, ‘Palestinian Hagiography (Fourth–Eighth Centuries)’, in S. Efthymiadis (ed.), *The Ashgate Research Companion to Byzantine Hagiography*, vol. 1: *Periods and Places*, Ashgate Research Companions (Farnham and Burlington, 2011), pp. 199–226, at p. 212.

^11^
Høgel, *Symeon Metaphrastes*, esp. p. 185. For the edition of the old *Life of St Matrona of Perge* (*BHG* 1221), see *Acta Sanctorum* [hereafter *AASS*] Nov. III (1910), pp. 790–813. For the date of composition of the old version and a discussion of the possibility that this text was written by a monk at Bassianos’ monastery, see H. Delehaye, ‘De Sancta Matrona’, *AASS* Nov. III (1910), pp. 786–823, at pp. 789–90; J. Featherstone and C. Mango, ‘Life of St. Matrona of Perge’, in Talbot (ed.), *Holy Women of Byzantium*, pp. 13–64, at pp. 13–16. For the edition of the Metaphrastic version of the *Life of St Matrona of Perge* (*BHG* 1222), see *AASS* Nov. III (1910), pp. 813–22.

^12^
For this narrative technique applied to the *Life of Mary of Egypt*, see S. Efthymiadis, ‘Introduction’, in Efthymiadis (ed.), *The Ashgate Research Companion*, vol. 2, pp. 1–15, at p. 7; C. Messis, ‘Fiction and/or Novelisation in Byzantine Hagiography’, in Efthymiadis (ed.), *The Ashgate Research Companion*, vol. 2, pp. 313–41, at pp. 329–30. A similar structure is also found in the tenth‐century *Life of St Theoktiste of Lesbos* by Niketas Magistros (*BHG* 1723–4), which is largely based on the *Life of Mary*, see *Life of Theoktiste of Lesbos*, *AASS* Nov. IV (1925), pp. 224–33.

^13^

*Life of Mary of Egypt*, cc. 2–3, *PG* 87, cols 3697–3700.

^14^

*Life of Mary of Egypt, c. 3, PG* 87, col. 3700: Ἵνα γνῷς […] πόσαι εἰσὶ καὶ ἄλλαι πρὸς σωτηρίαν ὁδοί. English trans. Kouli, ‘Life of St. Mary of Egypt’, p. 72.

^15^
This conception of ascetic life goes back to the *Life of Antony*, in which Antony is presented as practising ascetic discipline first near his own house and then in the tombs some distance from his town, before eventually retreating to the desert, see Athanasios of Alexandria, *Life of Antony*, cc. 3, 8, ed. Bartelink, pp. 134–8 and 156–8.

^16^

*Life of Mary of Egypt*, c. 5, *PG* 87, col. 3701.

^17^

*Life of Mary of Egypt*, c. 6, *PG* 87, col. 3701: Τῆς δὲ πύλης τοῦ μοναστηρίου ἀνοιγομένης μηδέποτε, ἀλλὰ ἀεὶ κεκλεισμένης καὶ ποιούσης τoῖς μοναχοῖς τὴν ἄσκησιν ἀνενόχλητον· οὐδὲ γὰρ ἦν ἀνοῖξαι, εἰ μή που μοναχὸς διά τινα χρείαν ἀναγκαίαν παρέβαλλεν· ἔρημος γὰρ ἦν ὁ τόπος, καὶ τοῖς ἐκ γειτόνων πλείοσι μοναχοῖς οὐκ ἀνεπίβατος δὲ μόνον ἀλλὰ καὶ ἄγνωστος. English trans. Kouli, ‘Life of St. Mary of Egypt’, p. 74, slightly modified.

^18^

*Life of Mary of Egypt*, c. 7, *PG* 87, col. 3704: Τὸν γὰρ Ἰορδάνην εὐθὺς περαιούμενοι, μακρὰν ἀπ᾿ ἀλλήλων ἕκαστος ἐχωρίζετο, καὶ πόλιν εἶχε τὴν ἔρημον. English trans. Kouli, ‘Life of St. Mary of Egypt’, pp. 74–5. Cf. the relevant passage in the *Life of Antony*, c. 14, ed. Bartelink, pp. 172–4. For the relationship between the desert and the city in early Christian literature, especially in hagiographical texts (including the *Life of Antony* – yet without any reference to the *Life of Mary of Egypt*), see C. Rapp, ‘Desert, City, and Countryside in the Early Christian Imagination’, in J. Dijkstra and M. van Dijk (eds), *The Encroaching Desert: Egyptian Hagiography and the Medieval West* (Leiden and Boston, 2006), pp. 93–112; A. Papaconstantinou, ‘The Desert and the City: The Rhetoric of Savagery and Civilization in Some Early Byzantine Narratives’, in K. Durak and I. Jevtić (eds), *Identity and the Other in Byzantium: Papers from the Fourth International Sevgi Gönül Byzantine Studies Symposium* (Istanbul, 2019), pp. 83–92; R. Stearn, *Historiography and Hierotopy: Palestinian Hagiography in the Sixth Century a.d*., Gorgias Studies in Classical and Late Antiquity 27 (Piscataway, 2020), esp. at pp. 100–5, 117–21.

^19^

*Life of Mary of Egypt*, c. 9, *PG* 87, col. 3704: ἔχων ἀεὶ τῆς κινήσεως τὸν τόνον ἀνένδοτον. English trans. Kouli, ‘Life of St. Mary of Egypt’, pp. 75–6.

^20^

*Life of Mary of Egypt*, c. 9, *PG* 87, col. 3704: κατὰ τὴν [ἐνδοτέραν] ἔρημον, cf. col. 3705 (c. 11). English trans. Kouli, ‘Life of St. Mary of Egypt’, pp. 75–6. The phrase ἐνδοτέρα/ἐσωτέρα ἔρημος also appears in the *Life of Antony*, c. 49.4, ed. Bartelink, p. 268.

^21^

*Life of Mary of Egypt*, c. 10, *PG* 87, col. 3705. English trans. Kouli, ‘Life of St. Mary of Egypt’, p. 76.

^22^
Cf. L. Coon, *Sacred Fictions: Holy Women and Hagiography in Late Antiquity* (Philadelphia, 1997), at pp. 87–9; P.C. Miller, ‘Is There a Harlot in This Text? Hagiography and the Grotesque’, *Journal of Medieval and Early Modern Studies* 33.3 (2003), pp. 419–35, at p. 426; A. Andreou, ‘Many Faces, a Single Pair: Rewriting Mary and Zosimas in the Legend of Mary of Egypt’, in S. Constantinou and C. Høgel (eds), Metaphrasis*: A Byzantine Concept of Rewriting and Its Hagiographical Products*, The Medieval Mediterranean 125 (Leiden and Boston, 2021), pp. 107–38, at p. 134.

^23^
See *Life of Mary of Egypt*, c. 16, *PG* 87, col. 3709.

^24^

*Life of Mary of Egypt*, c. 21, *PG* 87, col. 3712: Ἐγὼ τοίνυν […] καταπλήττομαι πῶς ὑπήνεγκεν ἡ θάλασσα τὰς ἐμὰς ἀσωτείας· […]. Ἀλλ᾿ ὡς ἔοικεν ὁ Θεὸς τὴν ἐμὴν ἐζήτει μετάνοιαν. English trans. Kouli, ‘Life of St. Mary of Egypt’, p. 81.

^25^
S. Efthymiadis, ‘The Sea as Topos and as Original Narrative in Middle and Late Byzantine Hagiography’, in N. Jaspert, C.A. Neumann and M. di Branco (eds), *Ein Meer und seine Heiligen: Hagiographie im Mittelalterlichen Mediterraneum*, Mittelmeerstudien 18 (Paderborn, 2018), pp. 109–21, at p. 112.

^26^
Kouli, ‘Life of St. Mary of Egypt’, p. 82 n. 48.

^27^
Cf. B. Ward, *Harlots of the Desert: A Study of Repentance in Early Monastic Sources* (London and Oxford, 1987), p. 33; Coon, *Sacred Fictions*, p. 87.

^28^
See *Life of Mary of Egypt*, cc. 26–9, *PG* 87, cols 3716–17.

^29^
For the conventional role of an average woman and the perception of prostitution in the male‐dominated society of Byzantium, see A.‐M. Talbot, ‘Women’, in G. Cavallo (ed.), *The Byzantines*, trans. T. Dunlap, T.L. Fagan and C. Lambert (Chicago and London, 1997), pp. 117–43. Repr. in A.‐M. Talbot (ed.), *Women and Religious Life in Byzantium*, Variorum Collected Studies 733 (Aldershot *et al*., 2001), I, pp. 117–43, esp. at pp. 117–18, 129–30; D. Ariantzi, ‘Byzantinische Prostituierte. Zwischen Marginalisierung und Reintegration in die Gesellschaft’, *Byzantion* 91 (2021), pp. 1–45 (with further references).

^30^

*Life of Mary of Egypt*, c. 28, *PG* 87, cols 3716–17 (wanderings in the desert): δεκαεπτὰ ἔτη εἰς ταύτην περιῆλθον τὴν ἔρημον, θηρσὶν ἀνημέροις ταῖς ἀλόγοις ἐπιθυμίαις πυκτεύουσα, cf. col. 3709 (c. 18: wanderings in the city). English trans. Kouli, ‘Life of St. Mary of Egypt’, pp. 80, 85.

^31^
According to her *Life* (*BHG* 1723–4), Theoktiste lived as an ascetic on the deserted island of Paros for thirty‐five years, see *Life of Theoktiste of Lesbos*, c. 16, *AASS* Nov. IV (1925), p. 229. Cf. I. Nilsson, ‘The Same Story, but Another: A Reappraisal of Literary Imitation in Byzantium’, in E. Schiffer and A. Rhoby (eds), *Imitatio – aemulatio – variatio: Akten des internationalen wissenschaftlichen Symposions zur byzantinischen Sprache und Literatur (Wien, 22.–25. Oktober 2008)*, Veröffentlichungen zur Byzanzforschung 21. Denkschriften der phil.‐hist. Klasse 402 (Vienna, 2010), pp. 195–208.

^32^
Antony’s almost seventy‐year stay in the desert is discussed in the greater part of his *Life*, see Athanasios of Alexandria, *Life of Antony*, cc. 11–92, ed. Bartelink, pp. 164– 372. For the 97‐year stay of Paul of Thebes in the desert as described in the Greek text (*BHG* 1466), see *Life of Paul of Thebes*, cc. 5–16, ed. K.S.T. Corey, ‘The Greek Versions of Jerome’s *Vita Sancti Pauli*’, in W.A. Oldfather *et al*. (eds), *Studies in the Text Tradition of St. Jerome’s* Vitae Patrum (Urbana, 1943), pp. 158–72.

^33^
Cf. R. Betancourt, *Byzantine Intersectionality: Sexuality, Gender, and Race in the Middle Ages* (Princeton and Oxford, 2020), pp. 5–14 (with a discussion of her iconography), who – yet without any reference to Mary’s wanderings in the desert – recently argued that her dark skin is a masculine feature, which Mary shares with the nuns in monks’ garments or other ‘manly women’. Still, this is not attested in the case of Matrona, examined in the second part of this study, whose hagiographer emphatically declares the white colour of her skin (τὸ δὲ χρῶμα λευκόν): *Life of Matrona of Perge*, c. 14, *AASS* Nov. III (1910), p. 797.

^34^
For the Byzantine ideal, according to which holy women had to escape their femininity and emulate men, see, for example, Talbot, ‘Women’, in *eadem*, *Women and Religious Life*, I, p. 118; Constantinou, *Female Corporeal Performances*, esp. pp. 90–106; N. Delierneux, ‘The Literary Portrait of Byzantine Female Saints’, in Efthymiadis (ed.), *The Ashgate Research Companion*, vol. 2, pp. 363–86.

^35^
Cyril of Skythopolis, *Life of Kyriakos* p.233.4–32, p.234.1–23, ed. E. Schwartz, *Kyrillos von Skythopolis*, Texte und Untersuchungen zur Geschichte der altchristlichen Literatur 49.2 (Leipzig, 1939), pp. 222–35, at pp. 233–4.

^36^
Cf. B. Flusin, ‘Le serviteur caché ou le saint sans existence’, in P. Odorico and P.A. Agapitos (eds), *Les Vies des Saints à Byzance: Genre littéraire ou biographie historique? (Actes du IIe colloque international philologique ‘Hermeneia’, Paris, 6–7–8 juin 2002)*, Dossiers Byzantins 4 (Paris, 2004), pp. 59–71; Delierneux, ‘The Literary Portrait’, p. 371.

^37^
For example, Antony left the desert at least for a while and went to Alexandria to defend the Orthodox faith against Arianism and interact with the people of the city, see Athanasios of Alexandria, *Life of Antony*, cc. 69–71, ed. Bartelink, pp. 314–20.

^38^

*Life of Mary of Egypt*, c. 26, *PG* 87, col. 3716: Γέγονα […] κατὰ ταύτην τὴν ἔρημον, καὶ ἐξ ἐκείνου μέχρι τῆς σήμερον *ἐμάκρυνα φυγαδεύουσα, καὶ ἐν ταύτῃ αὐλίζομαι, προσδεχομένη* τὸν Θεόν μου [cf. Psalm LIV (LV).7–8]. English trans. Kouli, ‘Life of St. Mary of Egypt’, p. 85.

^39^
Both Mary and Zosimas are commemorated as saints not only in the *Life* examined here, but also in the tenth‐century *Synaxarion of Constantinople*, ed. H. Delehaye, *Synaxarium ecclesiae Constantinopolitanae: Propylaeum ad Acta Sanctorum Novembris* (Brussels, 1902), pp. 577–80 (on 1 April).

^40^
See *Life of Mary of Egypt*, cc. 40–1, *PG* 87, col. 3725.

^41^
Ward, *Harlots of the Desert*, pp. 32–4; Coon, *Sacred Fictions*, p. 91; Kouli, ‘Life of St. Mary of Egypt’, p. 65; G. Leiser, *Prostitution in the Eastern Mediterranean World: The Economics of Sex in the Late Antique and Medieval Middle East* (London and New York, 2017), pp. 12–13; Ariantzi, ‘Byzantinische Prostituierte’, p. 6.

^42^
Cf. B. Caseau, ‘Christian Bodies: The Senses and Early Byzantine Christianity’, in L. James (ed.), *Desire and Denial in Byzantium (Papers from the Thirty‐First Spring Symposium of Byzantine Studies, University of Sussex, Brighton, March 1997)*, Society for the Promotion of Byzantine Studies 6 (Aldershot *et al*., 1999), pp. 101–9, at p. 102: ‘most of the prominent figures among the saints, such as the stylites, were not offered as an example to follow but as one to admire’. In my view, this is true not only for the case of ‘exotic’ ascetics, especially stylites and holy fools, but also for the unique case of Mary of Egypt.

^43^

*Life of Matrona of Perge*, c. 1, *AASS* Nov. III (1910), p. 790: τὸν τῆς μακαρίας καὶ ἁγίας Ματρώνης ψυχωφελῆ βίον, γυναικὸς τὰ ἀνδρῶν ἁγίων μεταξὺ ἀνδρῶν ἐγκρατῶν […] ἐπιδειξαμένης καὶ τὰ τῶν ἐν ἐρημίαις κατορθούντων κατορθωσάσης. English trans. Featherstone and Mango, ‘Life of St. Matrona of Perge’, p. 18, slightly modified.

^44^

*Life of Matrona of Perge* by Symeon Metaphrastes, c. 1, *AASS* Nov. III (1910), p. 813: Ματρώνης […] ἀνδράσι μὲν ἁμιλληθείσης τὴν ἀρετήν, παρενεγκούσης δὲ τούτους ὑπερφυέσι τοῖς κατορθώμασιν. English trans. K. Bennasser, ‘Gender and Sanctity in Early Byzantine Monasticism: A Study of the Phenomenon of Female Ascetics in Male Monastic Habit, with a Translation of the Life of St. Matrona’, Ph.D. thesis, Rutgers University (1984), p. 119, slightly modified.

^45^
For women in men’s clothing and their efforts to escape femininity, see, most recently, Betancourt, *Byzantine Intersectionality*, pp. 89–120 (with references to previous literature); Papavarnavas, *Gefängnis als Schwellenraum*, esp. at pp. 105–11.

^46^
Delehaye, ‘De Sancta Matrona’, *AASS* Nov. III (1910), pp. 786–823; Featherstone and Mango, ‘Life of St. Matrona of Perge’, pp. 13, 15; Constantinou, *Female Corporeal Performances*, p. 107. To the best of my knowledge, the only comparable example of a woman travelling from one city to another (yet not in male clothing) is that of Melania the Younger, which has been thoroughly studied by E.A. Clark, *Melania the Younger: From Rome to Jerusalem*, Women in Antiquity (New York, 2021).

^47^
Featherstone and Mango, ‘Life of St. Matrona of Perge’, p. 14.

^48^

*Life of Matrona of Perge*, c. 2, *AASS* Nov. III (1910), p. 791; and *Life of Matrona of Perge* by Symeon Metaphrastes, c. 3, *AASS* Nov. III (1910), p. 813.

^49^

*Life of Matrona of Perge*, c. 4, *AASS* Nov. III (1910), p. 792: ἡ τοῦ Θεοῦ χάρις, cf. p. 794 (c. 8). English trans. Featherstone and Mango, ‘Life of St. Matrona of Perge’, p. 22.

^50^

*Life of Matrona of Perge*, c. 4, *AASS* Nov. III (1910), p. 792; and *Life of Matrona of Perge* by Symeon Metaphrastes, cc. 6–7, *AASS* Nov. III (1910), p. 814.

^51^

*Life of Matrona of Perge*, c. 8, *AASS* Nov. III (1910), p. 794: ὁδηγίᾳ Θεοῦ. English trans. Featherstone and Mango, ‘Life of St. Matrona of Perge’, p. 27.

^52^
On this matter, see Constantinou, ‘Holy Actors and Actresses’, pp. 345–6; Delierneux, ‘The Literary Portrait’, p. 369, incl. n. 13.

^53^

*Life of Matrona of Perge*, c. 7, *AASS* Nov. III (1910), p. 794: στενάζουσα ἔλεγεν· Οἴμοι τῇ ἀθλίᾳ, ὅτι ἀπορρίπτομαι ὡς ἀναξία· οἴμοι τῇ ταλαιπώρῳ, ὅτι ἐγνώσθη ὅ εἰμι, καὶ οὐκέτι ὡς ἀδελφὸς τοῖς ἀδελφοῖς διαλέγομαι, οὐκέτι ὡς εὐνοῦχος νοοῦμαι καὶ Βαβυλᾶς φωνοῦμαι, ἀλλὰ πάλιν γυνὴ ὁρῶμαι καὶ Ματρῶνα καλοῦμαι. English trans. Featherstone and Mango, ‘Life of St. Matrona of Perge’, p. 26.

^54^
The omission of this outburst from the Metaphrastic version is intriguing, especially if one considers that in many other cases Symeon Metaphrastes chooses to focus on emotional episodes; see, most recently, L. Franco, ‘Psychological Introspection and the Image of Sanctity in the Metaphrastic *Menologion*’, in Constantinou and Høgel (eds), Metaphrasis, pp. 249–69.

^55^
Beside male figures, there are also several female characters (especially in the first part of the narrative) who take on a secondary, yet important and beneficial role in the story of Matrona. This topic is thoroughly examined by E. Catafygiotu‐Topping, ‘St. Matrona and Her Friends: Sisterhood in Byzantium’, in J. Chrysostomides (ed.), *Kathegetria: Essays Presented to Joan Hussey for Her 80th Birthday* (Camberley, 1988), pp. 211–24.

^56^

*Life of Matrona of Perge*, c. 11, *AASS* Nov. III (1910), p. 796.

^57^
For the city of Paneas, see J. Wilkinson, *Jerusalem Pilgrims before the Crusades* (Jerusalem, 1977; rev. 2002), p. 167. Cf. Featherstone and Mango, ‘Life of St. Matrona of Perge’, p. 34 n. 62.

^58^

*Life of Matrona of Perge*, c. 15, *AASS* Nov. III (1910), p. 798: Ἐν μιᾷ οὖν τῶν ἡμερῶν τὴν νυκτερινὴν ψαλμῳδίαν ποιουμένης αὐτῆς, ἀντέψαλλον οἱ δαίμονες μάλα προθύμως. Ἤκουεν γὰρ πολλῶν ἀνδρῶν φωνὰς ψαλλόντων, εἰς δειλίαν δὲ καταστᾶσα καὶ τῷ σημείῳ τοῦ σταυροῦ ἑαυτὴν τειχίζουσα, πληρώσασα τὴν ψαλμῳδίαν διελογίζετο ἐν ἑαυτῇ λέγουσα ὅτι· «ὁ μὲν τόπος ἔρημός ἐστιν, ὁ δὲ οἶκος βέβηλος, χωρίον ἐν τῷ τόπῳ τούτῳ οὐκ ἔστιν, παροδῖται ὧδε οὐ παρέβαλον, πόθεν ἄρα αἱ φωναὶ αὗται; English trans. Featherstone and Mango, ‘Life of St. Matrona of Perge’, p. 35, slightly modified.

^59^
Cf. Rapp, ‘Desert, City, and Countryside’, esp. pp. 93–8.

^60^
See Athanasios of Alexandria, *Life of Antony*, cc. 8–9, 12–14, ed. Bartelink, pp. 156–62, 166–74.

^61^

*Life of Matrona of Perge*, c. 16, *AASS* Nov. III (1910), p. 798: ἐχορήγει οὖν αὐτῇ ὁ τόπος ἐκεῖνος ἐξ ἐκείνου ὥσπερ φόρον τὴν ἡμερησίαν τροφὴν πληρεστάτην. English trans. Featherstone and Mango, ‘Life of St. Matrona of Perge’, p. 36.

^62^

*Life of Matrona of Perge*, c. 17, *AASS* Nov. III (1910), p. 799: οὐκ ἔστιν ὸ τόπος οὗτος ἄξιός σου· εἰδώλων γὰρ καὶ δαιμόνων οἰκητήριόν ἐστιν· δεῦρο εἰς τὴν Βηρυτόν, ὅτι πόλις ἐστὶ καλὴ καὶ φιλόξενος καὶ πάντας συγκροτοῦσα· τὰ γοῦν ένταῦθα ἐρημία ἐστίν, ὅτι τὰ ἐπιτήδεια οὐχ εὑρίσκονται. English trans. Featherstone and Mango, ‘Life of St. Matrona of Perge’, p. 36. The words of the demon are similar to those in the Metaphrastic version, see *Life of Matrona of Perge* by Symeon Metaphrastes, c. 20, *AASS* Nov. III (1910), p. 818.

^63^
A great difference between Matrona and Dometianos should be noted. Although Dometianos is also constantly on the move in pursuit of Matrona, there is no ascetic intention or motivation behind these movements, nor does he choose to live in ascetic or monastic confinement. Therefore, he does not improve spiritually.

^64^
Matrona also sees this sea journey as God’s will, cf. *Life of Matrona of Perge*, cc. 26, 28, *AASS* Nov. III (1910), pp. 802–3; and *Life of Matrona of Perge* by Symeon Metaphrastes, cc. 25, 27, *AASS* Nov. III (1910), p. 819.

^65^

*Life of Matrona of Perge* by Symeon Metaphrastes, c. 30, *AASS* Nov. III (1910), p. 820: τὸν τόπον […] ἐν δεξιᾷ μὲν τὴν θάλατταν ἔχοντα, ἑτέρωθι δὲ ἐκ γειτόνων παρέχοντα τὸ τοῦ Βασιανοῦ φροντιστήριον. English trans. Bennasser, ‘Gender and Sanctity’, p. 148, slightly modified.

^66^
See *Life of Matrona of Perge*, cc. 29, 51, *AASS* Nov. III (1910), pp. 803–4, 812.

^67^
The tenth‐century *Synaxarion of Constantinople* also makes no reference to the practice of wearing male attire at the convent, see *Synaxarion of Constantinople*, ed. Delehaye, pp. 203–5 (on 8 and 9 November). Cf. the illustration of Matrona in the *Menologion* of Basil II (*c*.1000), in which she is depicted in a nun’s habit: Biblioteca Apostolica Vaticana, Vat. gr. 1613, p. 169 (http://digi.vatlib.it/view/MSS_Vat.gr.1613).

^68^
See *Life of Matrona of Perge*, cc. 36, 41, 46, 50–2, *AASS* Nov. III (1910), pp. 806–7, 808–9, 810–11, 812–13.

^69^

*Life of Matrona of Perge* by Symeon Metaphrastes, c. 36, *AASS* Nov. III (1910), p. 822: λιποῦσα πρὸς τὸν ποθούμενον αὐτῇ Χριστὸν ἐξεδήμει, πράξεις καταλιποῦσα παρὰ πάντων μὲν διαφερόντως θαυμαζομένας, ὀλίγους δὲ πρὸς μίμησιν ὑπάγεσθαι δυναμένας. English trans. Bennasser, ‘Gender and Sanctity’, p. 154.

